# CrossFit participants’ experiences of balancing family, work, and fitness in a South African context

**DOI:** 10.3389/fpsyg.2026.1868541

**Published:** 2026-09-17

**Authors:** Cherissa O'Brien, Vicki Koen

**Affiliations:** School of Psychosocial Health, Community Psychosocial Health (COMPRES), Faculty of Health Sciences, North-West University, Potchefstroom, South Africa

**Keywords:** CrossFit, fitness, South Africa, work-family balance, work-life balance

## Abstract

**Introduction:**

Balancing family, work, and fitness is essential for overall well-being, yet navigating these competing demands presents a significant daily challenge. While work-family dynamics are well-documented, limited attention has been given to the integration of physical fitness into a balanced lifestyle. This study explored the experiences of CrossFit participants in balancing family, work, and fitness within a South African context.

**Methods:**

A qualitative, explorative-descriptive design was utilized. Purposive sampling was used to recruit eight participants of diverse ages, genders, and employment statuses from CrossFit boxes in Johannesburg, South Africa, with data collected via semi-structured interviews. Data were analyzed using thematic analysis.

**Results:**

Analysis yielded five main themes: intentional planning and routine; maintaining mental and physical well-being; flexibility and adaptability; consequences of imbalance; and systems of support.

**Discussion:**

The findings emphasize the key role of strategies such as planning, adaptability, and support in maintaining equilibrium across domains. These insights contribute to the broader discourse on work-family balance and underscore the need for targeted interventions that facilitate holistic balance.

## 1 Introduction

The World Health Organization (WHO) (2024) defines *physical activity* as any bodily movement that involves the use of skeletal muscles and requires energy expenditure. Engaging in physical activities offers substantial advantages for cardiovascular health, physical well-being, and mental acuity (World Health Organization (WHO), 2024). Having the available capacity to engage in physical activities and movements to complete everyday responsibilities and recreational activities and handle unexpected situations with enthusiasm and attentiveness, sufficient vitality, and without excessive exhaustion is often referred to as *fitness* or *physical fitness* (Farley et al., 2020). Fitness levels can be improved by *exercise*, which refers to a type of physical activity that is intentionally organized, structured, repetitive, and aimed at improving physical fitness, performance, or overall health (Saunders et al., 2020; World Health Organization (WHO), 2024). Exercise can be incorporated into people’s lifestyles in a variety of forms; however, one form that has gained rapid popularity as a fitness trend over recent years is CrossFit (Meier et al., 2023). CrossFit presents a compelling context for examining work-life balance due to its unique integration of high-intensity, time-efficient group workouts with a strong subcultural community. Unlike flexible solo exercise, CrossFit relies on scheduled class times, demanding physical effort, and communal involvement, requiring participants to negotiate strict time boundaries between work responsibilities, personal life, and training commitments (Gavanda et al., 2022).

To theoretically anchor how working adults negotiate these competing domains, this study utilizes Conservation of Resources (COR) theory (Hobfoll et al., 2018). COR theory asserts that individuals strive to acquire, protect, and foster valued resources including time, physical energy, emotional bandwidth, and social support. Within this framework, chronic work and family demands threaten resource depletion (leading to strain and burnout), whereas structured high-intensity exercise and community involvement act as mechanisms for resource generation and psychological recovery (Ünal and Ekemen, 2026).

### 1.1 CrossFit

CrossFit workouts are daily exercise routines designed to incorporate metabolic exercises, including the use of free weights, body weight, running, and cycling. In CrossFit lingo, these routines are collectively referred to as the “workout of the day” (WOD). A typical CrossFit class has a structured sequence that includes a warm-up, a strength component, and the WOD. Unlike self-directed gym exercise, these structured one-hour sessions are conducted in a group setting under the guidance of a certified coach, fostering a supportive community culture and strong peer accountability. WOD routines change daily, incorporate intense exercises from gymnastics, weightlifting, and cardiovascular activities (Gavanda et al., 2022), are characterized by constant variation and aim to assess power, strength, and cardiovascular endurance, with the primary objective of completing the assigned task as swiftly as possible, or to achieve the highest number of repetitions or rounds within a specified time frame (Meier et al., 2023) of approximately 1 h (Zeitz et al., 2020). The WODs involve repetitive performance of exercises for a designated duration, often with limited or no rest periods (Gavanda et al., 2022).

CrossFit has rapidly emerged as one of the fastest-growing training methods, with more than 15,000 affiliated training centers and 5 million athletes. It was also listed among the top 20 global fitness trends of 2022 (Meier et al., 2023). In contemporary South Africa, the popularity of CrossFit as a form of exercise, including exercise for families, has also been on the rise (Simpson et al., 2017). While numerous case reports highlight how CrossFit enhances health and transforms lifestyles, CrossFit’s associations with balancing fitness, family, and work are of particular interest for the purpose of this study.

Known for its high-intensity and varied workouts, CrossFit not only enhances physical fitness but also provides mental health benefits through its supportive community and stress-relieving properties (Gavanda et al., 2022). In addition, CrossFit’s flexibility makes it a viable option for family participation, physical health promotion, and family cohesion (Martinho et al., 2024; Rios et al., 2024). Moreover, as sessions are usually 1 h long and feature workouts consisting of constantly varied functional movements performed at a high intensity (Claudino et al., 2018), this approach can be advantageous to individuals seeking to manage their professional, personal, and physical fitness obligations simultaneously, as it allows for the optimisation of time compared to more conventional forms of training (Martinho et al., 2024) Similarly, CrossFit allows for scalable workouts and is inclusive in nature, offering a potential solution for busy individuals looking to incorporate fitness into their lives (Martinho et al., 2024)From a COR perspective, CrossFit represents a time-efficient investment of personal energy that yields multi-domain resource returns such as heightened vigor, stress relief, and social capital which can then be reinvested back into work and domestic roles (Hobfoll et al., 2018).

While work and family life are distinct yet interconnected spheres that positively and negatively influence each other (Gragnano et al., 2020), the potential value of CrossFit is highlighted by the increasing difficulty of managing the modern demands associated with work environments and professional and family responsibilities (Abdou et al., 2024). Thus, CrossFit offers a unique approach to balancing fitness, family, and work demands.

### 1.2 Work-family balance and conflict

Work-family balance entails discovering a balanced routine and self-perceived harmony in one’s ability to effectively blend one’s work with one’s family obligations, personal pursuits, and ambitions outside of work (Gragnano et al., 2020). Keeney et al. (2013) delineated eight non-work spheres pertinent to work-life balance: education, health, recreation, social connections, romantic partnerships, family, household administration, and community engagement. The importance individuals place on these domains varies from person to person, and the hierarchical importance of these life domains may evolve over time owing to shifts in personal interests and life circumstances (Daks and Rogge, 2020).

Maintaining a balance between these spheres minimizes stress-induced consequences, including psychological strain and emotional depletion. Under COR theory, psychological strain occurs when individuals experience a net loss of resources, the threat of resource loss, or a failure to gain resources following significant resource investment (Hobfoll et al., 2018). Additionally, an enhanced work-family balance not only cultivates job satisfaction, − performance, and - dedication, but also contributes to contentment in personal - and family life (Gragnano et al., 2020). When key personal resources such as self-efficacy and psychological resilience generated in one domain spill over to enhance performance in another, work-family enrichment occurs (Lu et al., 2021).

However, when demands or pressures from one domain, such as work, interfere with fulfilling responsibilities in the other, such as family, or personal health, *Work–Family Conflict* (WFC) occur. WFC can manifest in three forms: time-based (work encroaches on family time); strain-based (stress from one domain spills over into the other); and behavior-based (role expectations conflict) (Huo and Jiang, 2023). In COR terms, WFC represents a core resource drain scenario, where competing domain demands create a resource loss spiral (Hobfoll et al., 2018).

WFC is closely linked to work stress and its impact on employees’ well-being and prolonged exposure to WFC can lead to burnout and emotional – and psychological distress (Sirgy et al., 2019), as the ongoing stress of managing work and family roles can contribute to anxiety, tension, and a feeling of being overwhelmed (Dodanwala et al., 2022). Thus, maintaining a work-family balance is crucial for mental well-being, as chronic stress in either domain can significantly impact psychological health (Gragnano et al., 2020).

## 2 Rationale

Literature highlights the relevance of effectively balancing work, family, and fitness, especially to support mental health. Yet there remains a gap in current literature regarding the intersection of physical fitness, particularly CrossFit, with work and family life. The complexity of balancing these three domains has not been thoroughly explored, especially in contexts like South Africa. This gap is significant given the unique socio-economic challenges and family dynamics present in South Africa, which may influence how individuals manage these aspects of their lives. In response, this study aimed to explore and describe CrossFit participants’ experiences of balancing family, work, and fitness in a South African context.

## 3 Methods

### 3.1 Research design

A qualitative explorative-descriptive research design was applied. According to Hunter et al. (2019), exploratory research aims to shed light on how a particular phenomenon is expressed to reveal comprehensive characteristics of poorly understood phenomena. Descriptive research focuses on providing a detailed account of a phenomenon’s significance to generate a comprehensive understanding thereof from participants’ perspective (Hunter et al., 2019). The design was considered suitable for this study to gain a more comprehensive understanding of how individuals who engage in CrossFit can balance their family, work and fitness roles.

### 3.2 Population

The population included adult family members who engaged in CrossFit in Johannesburg, South Africa and were either formally employed or self-employed. According to “CrossFit” (2023), Johannesburg has the highest number of registered CrossFit boxes in South Africa, totalling 69 boxes. A further rationale behind selecting participants from Johannesburg is the substantial disparity in work dynamics, lifestyle pace, and environmental stressors when comparing individuals in metropolitan areas to those in smaller towns (Ganiyu et al., 2020). As South Africa’s primary economic hub, Johannesburg is characterized by a fast-paced environment where individuals frequently face extended working hours, long daily commutes, and heightened workplace performance expectations (Gobind, 2018; Ganiyu et al., 2020). These heightened lifestyle and occupational demands create a compelling context for investigating how working adults structure their daily routines to maintain a balance between family life, professional responsibilities, and high-intensity exercise.

### 3.3 Sampling

Participants were recruited using a purposive sampling strategy (Boeije, 2014) across two affiliated CrossFit boxes in Johannesburg. Recruitment advertisements were distributed both as physical posters displayed in high-traffic areas of the facilities and as digital flyers shared via the boxes’ official social media groups and member communications. To be eligible, participants had to be adults residing in Johannesburg, formally employed or self-employed, residing in a family unit, and actively participating in CrossFit for at least 1 year. Potential participants contacted the researcher directly via email or telephone in response to the advertisements. Screening for eligibility was conducted through a brief preliminary screening to confirm employment status, family structure, and training tenure.

An independent person provided eligible individuals with an information participant sheet and consent form prior to scheduling interviews. The final sample comprised eight participants (see Table 1 for a demographic profile). Sample size was guided by principles of code and meaning saturation (Hennink and Kaiser, 2022). Data collection and analysis occurred concurrently; allowing saturation to be continuously monitored using a visual saturation grid tracking the emergence of new codes across sequential interviews. Code saturation, the point at which no additional unique codes were identified, was observed by the sixth interview. Two additional interviews were conducted to ensure meaning saturation, confirming that existing conceptual categories regarding work-life-fitness boundaries were fully elaborated with rich, nuanced participant descriptions without yielding novel conceptual directions.

**Table 1:** Demographic profile of participants.

Participant	Age	Gender	Number of children	Relationship status	Employment status	Working hours per day	Time spent at CrossFit (daily)
1	29	Female	0	Engaged	Formally employed	9 h	1 h
2	34	Female	0	Married	Formally employed	9 h	1 h
3	39	Female	0	Married	Formally employed	9 h	1 h
4	39	Male	0	Married	Formally employed	9 h	1 h
5	33	Male	0	Married	Self employed	8 h	1–2 h
6	20	Female	0	Single	Formally employed	4 h	1–2 h
7	55	Female	1	Married	Formally employed	8 h	1 h
8	32	Female	2	Married	Formally employed	Flexible hours	1 h

### 3.4 Procedure

The study received institutional approval and goodwill permission from the two participating CrossFit boxes. The owners of the CrossFit boxes acted as both gatekeepers and mediators in communicating with participants. Written informed consent was obtained from all participants prior to data collection, which included explicit permission for audio recording. Participants received a R150 Sportsman’s Warehouse voucher upon completion of the interview as a token of appreciation.

### 3.5 Data collection

Semi-structured interviews were held in person at the respective CrossFit boxes at a time that was convenient for the participants, in a room which was private, quiet and had available seating. All interviews were conducted by the first author to maintain consistency and minimize interviewer bias.

The interviews lasted between 45 and 65 min (averaging approximately 50 min) and were audio-recorded using a digital audio recorder. The interview schedule consisted of the following questions:

1. How do you experience balancing your family, work, and fitness?
2. How do you prioritize and allocate time between your family, work, and fitness? Please share examples.
3. What strategies or techniques have you used to effectively balance your family, work, and fitness? Please share examples.
4. Have you experienced any challenges in balancing your family, work, and fitness commitments? If so, please share examples and explain how you managed them.
5. What role has balancing family, work, and fitness played in your overall well-being? Please share examples.

To encourage depth and rich description, flexible probing strategies were utilized (Roberts, 2020). Probes included clarifying questions (e.g., “Can you elaborate on what you mean by…”), elaboration requests (e.g., “Walk me through a specific time when that happened…”), and emotional and reflective prompts to explore underlying boundary tensions between work, family, and CrossFit commitments.

### 3.6 Trustworthiness

The study’s implementation of the criteria outlined by Lincoln and Guba (1985) is detailed in Table 2.

**Table 2:** Measures to ensure trustworthiness.

Criteria	Measures
Credibility	Reflexivity: Throughout the research process, the researcher reflected honestly and critically on herself and the research process through reflexive journaling. The researcher employed verbatim transcription and took field notes for the purpose of reflexivity and an audit trail. Co-coding: An experienced co-coder independently analysed data.
Dependability	Detailed description: To ensure consistency and repeatability in the study, a comprehensive account of the methodology and procedures is provided. Co-coding (see earlier description). An audit trail and field notes were kept by the researcher, consisting of thorough storage and documentation pertaining to all aspects of the study.
Confirmability	Reflexivity and audit trail (see earlier description).
Transferability	Detailed description (see earlier description).

### 3.7 Data analysis

The data was analysed using Braun and Clarke (2006) six-step thematic analysis framework. Data was managed manually without the use of qualitative data analysis software. To ensure dependability and maintain a transparent audit trail, the analysis was conducted systematically across the following six phases:

1. Data familiarization: The first author transcribed all audio recordings verbatim. Both the first author and a co-coder read and re-read the printed physical transcripts while listening to the audio files to gain a deep understanding of the narratives. Initial observational notes were written directly on the transcript and recorded.
2. Generation of initial codes: Initial coding was conducted manually (without qualitative data analysis software, using hard-copy transcript highlighting and systematic code categorization), following an inductive approach to allow themes to emerge naturally from the data. Coding disagreements were resolved through structured dialog and renegotiation until consensus was reached regarding code definitions and scope. The first author then manually applied the refined coding framework to the remaining transcripts.
3. Identification of themes: The initial codes were systematically reviewed to identify broader patterns leading to the development of themes and some sub-themes.
4. Review of themes: Themes were reviewed and refined against both the coded extracts and the full dataset. The first author and co-coder met to verify that the themes accurately reflected participant accounts and were distinct, cohesive, and fully supported by the empirical data.
5. Definition and naming of the themes: Each theme was clearly defined in alignment with the research objectives, ensuring that they provided meaningful insights into participants’ experiences.
6. Report writing: The first author integrated the aforementioned steps to concisely explain the findings.

A co-coder experienced in thematic analysis independently analysed the data. The first author and co-coder subsequently discussed their analysis to determine if they had reached a consensus regarding the identified themes. Any initial coding or thematic disagreements were resolved through collaborative discussion, re-examining raw transcript segments together, and refining code definitions until full consensus was achieved. All transcribed data, codebooks, and analytic decisions have been securely documented and archived to ensure an auditable process.

## 4 Findings

Table 3 offers an overview of the five main themes and sub-themes identified through the data analysis, followed by a detailed discussion. Direct quotes from participants are used to support the findings and, as a result, may contain grammatical inaccuracies.

**Table 3:** Overview of themes and sub-themes.

Themes	Sub-themes
Intentional planning and routine	–
Maintaining mental and physical well-being	–
Flexibility and adaptability	–
Consequences of imbalance between family, work and fitness domains	Experience of negative emotions Experience of role conflict.
Systems of support	Types of support systems The role of social support in sustaining long-term fitness goals.

### 4.1 Theme 1: intentional planning and routine

Participants emphasized that intentional planning and maintaining a structured routine contribute significantly to balancing family, work, and fitness domains. They highlighted the importance of early morning workouts, time blocking, scheduling, and maintaining discipline and consistency to achieve this balance.

Several participants stressed the importance of dedicating early morning hours to CrossFit training. This habit allowed them to start their day with a clear mind and a sense of accomplishment, helping to maintain consistency and minimize stress throughout the day. “I started training early in the day to ensure that I can have a productive day still at work without stressing too much about getting to workouts” Participant (P1). Other participants echoed similar sentiments: “I do find that if I do not exercise at five in the morning, then something happens at work or something just comes up during the day and it’s just too easy to make an excuse and not come at all” (P3); “I go to gym 5 a.m. So, I always try to do it at the start of the day, then I know I do not have to stress about it during the day” (P8).

Many participants emphasized the importance of blocking time for specific tasks and commitments, scheduling activities ahead of time, and sticking to a routine. This practice not only provided structure but also helped with accountability and balancing family, work, and fitness. P1 shared: “It’s just about getting at least half an hour in… planning the week ahead, setting the time, and going”. Moreover, P3 noted a technique that was used whereby: “I’ll set my calendar for the week, so I use Google calendars to just block out times.” P6 also described her technique of being able to schedule and stick to a routine by: “…I sit each Sunday and plan the week out…”.

In line with the importance of planning and routine, participants also highlighted the need for discipline and consistency in balancing family, work, and fitness: “… I’d say it’s tough but getting into the routine of it is makes it a lot easier and the discipline obviously.” (P5); “…I think if you can put a discipline in place and really follow through on that it becomes easier and because that becomes easier your cup fills up” (P5).

Analytically, this reliance on forward-planning reveals the invisible cognitive load and administrative labor required to manage a three-way boundary matrix (work, family, and fitness). Viewed through COR theory, proactive calendar blocking and disciplined routines function as resource-protective boundary tactics (Allen et al., 2021). By establishing rigid time boundaries early in the day, participants protect their temporal and cognitive resources from being depleted by unpredictable workplace or domestic demands later in the day (Hobfoll et al., 2018). Constantly coordinating schedules on Sunday evenings demonstrates that maintaining balance is an active, continuous cognitive process designed to prevent net resource loss.

### 4.2 Theme 2: maintaining mental and physical well-being

The interviews revealed the importance of training for both mental and physical well-being, including stress management, mental health, increased energy, daily functioning, and a sense of achievement and personal growth.

All participants emphasized how maintaining their fitness routines helped them manage stress and support their mental health: “…health is super important… working out is a massive priority for mental health, helping you be present and in a better headspace” (P1); “…you know, it can be sort of a more of a stress relieving thing” (P3); “a good stress relief…” (P4); “…if I do not go to CrossFit the stress is exaggerated exponentially…” (P5) “…exercise releases like all the right hormones” (P8).

Some participants experienced increased energy and daily functioning by incorporating training into their daily routines: “…getting a bout of fitness in so that be the hour in the morning fills my cup up which prepares me to be a better person during the day and make more sound decisions be a lot more motivated be a lot more productive” (P5); “We’re never sick” (P7).

Some participants mentioned that training provides them with a sense of achievement and personal growth: “…improved me as a human and I think as a husband…” (P5); “…accomplishing goals for yourself that’s more important than just work…” (P5).

Many participants stressed making training a priority for different reasons and needs: “…well, for me, I do it for fun. Um, so it’s, it’s a priority, but it’s also for me, it’s lekker.” (P4); “I cannot imagine coming home and not training…” (P7); “…but I’m very disciplined in making time to go gym.” (P8).

From an interpretive perspective, these accounts illustrate how intense physical exercise acts as a key mechanism for psychological detachment and role enrichment (Baktash and Pütz, 2025). Under COR theory, physical fitness training serves as an essential resource-generating activity rather than a resource-draining obligation. High-intensity physical exertion restores psychological bandwidth, generating personal energy and positive affect that can be reinvested into family and professional spheres a phenomenon known as work-fitness-family enrichment (Ünal and Ekemen, 2026). Furthermore, P5’s assertion that fitness made him a “better husband” and “fills my cup up” directly aligns with COR theory’s resource replenishment principle (Hobfoll et al., 2018), illustrating how investing temporal resources into self-care generates psychological resources that enhance interpersonal relationships rather than subtracting from them.

### 4.3 Theme 3: flexibility and adaptability

Participants highlighted the importance of flexibility and adaptability in their training schedules, including adapting to changing work environments and family demands, remote work and flexible job arrangements, and coping with unforeseen situations.

Most participants experienced the need to adjust their training schedules to accommodate shifting work and family demands: “…being able to spend time with family and friends in the evenings and over weekends. I just adjust my training program to be able to train in the mornings” (P1); “And family in week times…So they get time over the weekend, every other weekend more or less” (P4); “…work, in a sense, most of the time is, the time allocated is forced…” (P6); “…I think with work, you sort of go around the baby schedule. And also with gym, you go around when it’s going to be convenient…” (P8).

Several participants expressed gratitude for the flexibility afforded by remote work and flexible job structures. This flexibility allowed them to balance various roles more effectively: “I think my remote working circumstances…it makes it a lot easier to get around to everything…” (P1); “…I’m very lucky to work from home…” (P3); “I’m also quite fortunate that I work from home, so because of that I’m quite flexible in terms of my training” (P7); “…this year I’ve actually been working from home. So, it is more flexible” (P8).

Participants also discussed the impact of unexpected events, which often disrupted their balance: “…exercise is the thing that has to give when there’s work pressure or family stuff that needs to happen or whatever. Then exercise is the one that goes out the window, unfortunately” (P3); “…I’ve got my U. S. team phoning and saying can we have a conference call, and there goes my CrossFit workout for the afternoon…” (P7).

Participants emphasized the importance of maintaining consistency in their training while remaining flexible: “…even if I have to do work, so say I’ve got to do reports. So, say I’ve got two hours to do it, then I’ll sort of prioritize my time to do that, go for a run and then come back and do what I need to do…” (P2); “I think also making sure that you are joining a CrossFit gym or a CrossFit box that’s quite flexible with different times is also going to be important” (P7).

Through a critical structural lens, this adaptability relies heavily on job autonomy and socio-economic privilege (Lee, 2023). High boundary control and remote work arrangements allow knowledge workers to flex their schedules around fitness. In the language of COR theory, job autonomy acts as a crucial macro-resource that allows individuals to dynamically reallocate their time to protect wellness activities during acute stress events (Baum and Rau, 2024). However, when conflicts occur, personal exercise boundaries are consistently the most permeable and sacrificial compared to non-negotiable employment obligations, reflecting broader organizational power dynamics over personal time.

### 4.4 Theme 4: consequences of imbalance between family, work and fitness domains

Participants shared their experiences of how imbalances between fitness, work, and fitness can lead to the experience of negative emotions and role conflict.

#### 4.4.1 Experience of negative emotions

Participants frequently mentioned feelings of guilt and stress due to neglecting family and friends: “…you know, a lot of the time your effort and energy goes to predominantly one field and you do feel like you maybe lack to the other side” (P6); while P8 described, “…sense that you are always feeling you are lacking in areas. So, if you spend too long at the gym, you feel your family is coming in short. I do find you sort of feel guilty if you work too much…”. These emotions were compounded by external expectations. The emotional impact often manifested in feelings of inadequacy. P3 said: “…you feel like you are not good enough when you cannot do everything for everyone.”

#### 4.4.2 Experience of role conflict

Role conflict was common, with participants struggling to balance family, work and fitness. According to P6, “…work ended up being a non-negotiable”, leaving little room for personal time, while P3 admitted fitness was often sacrificed: “Whereas for those three areas of my life at least, fitness is the least important thing…”. Unexpected disruptions also heightened this conflict - P8 explained: “…husband goes hunting, he’s away for 5 days… I cannot go to gym at all…”. Participants felt overwhelmed by competing responsibilities, which led to frustration when they could not meet their own expectations. In addition, P6 shared: “…when I feel like I have not trained enough… or, you know, I have not seen family or friends… I feel unsettled and sort of uneasy…”.

Interpreted through COR theory, these experiences of guilt, emotional strain, and role conflict reflect a classic resource loss spiral (Hobfoll et al., 2018). When individuals invest substantial energy and time into one domain (e.g., work), they suffer a net resource deficit in other domains (e.g., family or fitness), triggering acute feelings of strain and psychological distress. This theme exposes the gendered asymmetries and internalized role expectations that govern boundary conflict (Aarntzen et al., 2020). Pervasive guilt particularly among female participants reflects the societal pressure of “intensive caregiving” norms, where personal exercise is evaluated against family duty. Women face heightened threat of resource loss because societal expectations demand uncompromised family investment, making personal time spent at the gym feel like an illegitimate resource expenditure. Furthermore, instances like P8’s inability to train while her spouse engaged in independent recreation highlight unequal domestic burdens, showing how role strain is compounded by traditional gender expectations.

### 4.5 Theme 5: systems of support

Participants emphasized the significance of strong support systems, including family, the CrossFit community, and friendships, in maintaining their long-term fitness goals. Two key sub-themes emerged: the different types of support systems and the critical role social support plays in sustaining long-term fitness commitments.

#### 4.5.1 Types of support systems

Participants emphasized the role of family, the CrossFit community, and friendships in maintaining balance. Family support was crucial in managing time and commitments: “We help each other get to the things we really want… it’s important for a family to have buy-in” (P3); P7 highlighted that their husband being “…into sport…” made it easier, while P8 mentioned: “…my gran watches the baby…” when extra work needed to be done.

The CrossFit community provided motivation and accountability. P4 shared: “I enjoy the community of CrossFit,” while P7 appreciated that having friends who train enabled a “balance” in their social life. Friendships also played a vital role in staying consistent with fitness goals, as P7 pointed out: “We surround ourselves with healthy friends that train as well.”

#### 4.5.2 The role of social support in sustaining long-term fitness goals

Participants found encouragement from loved ones to be key in achieving long-term fitness goals. For instance, P3 emphasized the importance of planning together, saying it was essential “…to prioritize and plan what you really want to do together”. P8 added a motivational element by looking forward to family involvement: “I’m looking forward to the day where the kids tag along and cheer for you at competitions.” This collective encouragement helped them stay committed to their multiple roles.

Overall, individual discipline is insufficient without instrumental support and social capital (Tough et al., 2022). Practical assistance, such as extended family caregiving, acts as a structural buffer that mitigates role strain. Through the theoretical lens of COR theory, support systems function as critical “resource caravans”—external social networks that aggregate and provide resources to individuals when their personal energy or time pools are depleted (Hobfoll et al., 2018). Practical support from family (instrumental resources) frees up time, while the subcultural social capital of the CrossFit box (emotional and relational resources) normalizes fitness pursuits. This allows participants to align personal health goals with their family identities rather than experiencing them as conflicting demands, thereby sustaining their health investments over the long term.

## 5 Discussion

The purpose of this study was to explore the experiences of working adults in balancing family, work, and fitness commitments within the context of CrossFit in Johannesburg, South Africa. To provide a robust analytical underpinning, the empirical findings are interpreted through the lens of Conservation of Resources (COR) theory (Demerouti, 2025). COR theory posits that individuals strive to obtain, retain, protect, and foster valued personal, social, and energy resources. In this study, work and family demands frequently act as resource-depleting stressors, whereas physical training serves as a mechanism for resource acquisition and psychological recovery.

The findings are discussed in relation to comparable studies. Regarding the importance of intentional planning and routine for a balance between family, work and fitness domains, several participants noted that exercising in the morning set a positive tone for the day, helped maintain consistency, minimized stress, and ensured exercise was not interrupted by other commitments. While this aligns with research by Rupali and Aruna (2020), which highlights the physiological and psychological benefits of morning exercise, and South African research on workplace wellness routines (du Plessis and van Zyl, 2026), it is crucial to question whether this strategy is equally accessible to all South Africans. In a country where many individuals commute long distances to work, some in public transport and face unpredictable work hours (Mlangeni et al., 2018), morning workouts may not be viable for everyone. While the sample comprised formally employed, middle-class adults with sufficient autonomy to select early morning training, this contrast highlights the structural privilege embedded within structured fitness routines. As noted in South African socioeconomic literature (Mlangeni et al., 2018), physical activity accessibility remains heavily stratified along class lines. This raises concerns about the socioeconomic disparities in fitness accessibility, where only those with flexible schedules or proximity to gyms can implement such strategies effectively.

Participants also emphasized intentional time management through time blocking and scheduling to prioritize fitness alongside work and family, as routines simplify decision-making and promote health, which is supported by Arlinghaus and Johnston (2018). Discipline and consistency were crucial in scheduling, as adhering to a schedule fosters balance between personal and professional responsibilities – a finding that Majini and Majini Jes Bella (2023) underpin. Available research notes that effective time management reduces stress, prevents burnout, and enhances work-life balance (Edú-Valsania et al., 2022; Chansaengsee, 2017). Strategies such as goal setting, prioritization, and boundary-setting further optimize time and improve satisfaction (Gautam and Jain, 2018). Through a COR theory perspective, structured time blocking acts as a resource-protective tactic (Allen et al., 2021), preventing work demands from invading personal health domains. By integrating discipline, routine, and time management, participants achieved fitness goals while maintaining balance in other life domains. However, it is worth considering whether such rigid approaches risk creating additional pressure rather than alleviating it. In the South African context, where work demands are often unpredictable and family obligations are culturally significant (Sandy et al., 2022), strict adherence to schedules may lead to stress when unexpected disruptions occur. Thus, while structure is beneficial, a balance between discipline and adaptability may be more sustainable.

The theme of the importance of physical exercise in maintaining mental and physical well-being highlights how participants view exercise as essential to their health. Participants indicated specific elements such as stress management, increased energy, personal growth, and making training a priority for different reasons and needs. Participants frequently described exercise as a tool for stress management and mental well-being, these being consistent with international literature demonstrating the psychological benefits of physical activity such as reducing symptoms of depression, anxiety, and stress through the release of endorphins and serotonin (Andermo et al., 2020; Mahindru et al., 2023; Martín-Rodríguez et al., 2024), yet the South African perspective adds a unique dimension. Given the high prevalence of workplace stress, financial strain and safety concerns in South Africa (Mlangeni et al., 2018), it is possible that exercise serves as not just a stress reliever, but a necessary coping mechanism for broader societal pressures. Viewed through COR theory, high-intensity exercise generates key personal resources such as physical stamina, emotional resilience, and self-efficacy which are subsequently reinvested into work and domestic roles (work-fitness-family enrichment) (Allen et al., 2021).

Crucially, the unique modality of CrossFit plays a specific role in this process. Unlike general solo gym workouts, CrossFit demands high physiological effort within a structured 60-min class format. This extreme physical focus forces rapid psychological detachment from work and domestic worries (Piszczek and McAlpine, 2026) offering an accelerated “reset” that standard moderate exercise may not yield. Many participants noted that training boosted energy and improved daily functioning, aligning with research that links exercise to enhanced cognitive function and overall health (da Silveira et al., 2020; Hallam et al., 2018; Wender et al., 2022). Participants also reported a sense of personal growth through fitness, reflecting research on physical activity as a path to self-improvement and mastery, strengthening self-esteem and life satisfaction (Biddle and Vergeer, 2020; Blom et al., 2021). This growth is highlighted by Participant 5: *“It has improved me as a human,”* which resonates with the concept of a “growth mindset,” which is the belief that abilities and intelligence can be developed through effort, learning, and perseverance, rather than being fixed traits as indicated by Dweck and Yeager (2019). For many participants, training was a priority for different reasons and needs. However, while prioritizing exercise can enhance life satisfaction, literature suggests there is a risk of exercise dependence if it interferes with other responsibilities (Weinstein and Szabo, 2023). With this in mind, balance and flexibility in fitness routines are regarded as crucial to prevent burnout and maintain long-term well-being. Overall, training was essential for participants’ mental and physical health, providing stress relief, increased energy, and personal growth. However, maintaining a flexible approach is necessary to avoid over-reliance on exercise for managing mental health.

The importance of flexibility and adaptability in training schedules is crucial for balancing domains. Participants emphasized the need to adjust their fitness routines to meet changing demands. Research supports this, showing that flexible approaches help sustain long-term fitness commitments (Rupali and Aruna, 2020). Many participants adapted by altering workout times, such as shifting to morning sessions to accommodate evening family time. Archer et al. (2024) found that adapting schedules reduces stress and increases motivation, contributing to better mental and physical outcomes. Remote work and flexible job arrangements were cited as advantages in maintaining regular training. Participants noted how working from home enabled them to plan their day more effectively around fitness. Studies confirm that remote workers are more likely to exercise regularly due to flexible schedules (da Silveira et al., 2020). It should be noted, however, that flexibility can blur work-life boundaries, making it essential to set clear limits to prioritize exercise (Andrade et al., 2019).

In the South African context, structural barriers may limit this flexibility. While some participants benefited from remote work options, many South Africans lack this privilege. High unemployment rates (South African Government, 2024) and the prevalence of shift work in industries such as healthcare and security often result in rigid schedules that directly conflict with fitness goals (Van Zyl, 2019). It is important to clarify that our empirical data reflect the lived realities of employed individuals who possess occupational autonomy; contrasting these findings with broader South African literature illustrates how occupational status buffers against rigid schedule constraints. This highlights the need for workplace policies that promote flexible scheduling, enabling more individuals to engage in regular physical activity.

Participants also shared how they managed unforeseen disruptions, such as family emergencies or last-minute work commitments, often rearranging workouts to maintain consistency. Flexibility, defined as adaptability, is a critical competency in today’s dynamic work environment, helping individuals stay consistent in their fitness routines despite challenges (Vylobkova and Heintz, 2023). Those who plan alternative workouts or remain flexible in their approach are better equipped to handle disruptions (Rupali and Aruna, 2020). While flexibility is key, maintaining consistency was seen as essential for long-term success. Participants emphasized that flexibility should not mean losing focus but rather adapting to stay on track. Research shows that incorporating flexibility into fitness routines leads to higher long-term adherence (Beshears et al., 2020). Additionally, fitness programs offering varied class schedules further support consistency (Gottschall and Hastings, 2023). Here, CrossFit boxes provide a distinct structural advantage over self-guided exercise: by offering multiple standardized class times throughout the day (e.g., 5 a.m., 6 a.m., 12 p.m., 5 p.m.), participants can flexibly swap training slots without losing the structure or coaching of a planned workout. Therefore, flexibility and adaptability are vital for balancing family, work, and fitness domains.

An imbalance between family, work and fitness domains was reported to often lead to emotional distress, particularly feelings of guilt and stress. Participants frequently expressed guilt when family or social obligations were neglected in favor of fitness or work. For instance, Participant (P)6 discussed the challenge of distributing energy equally across life domains, while P8 shared feelings of guilt for not meeting expectations in multiple areas. These emotional struggles align with the research by Isa and Indrayati (2023), who found that role conflict often results in emotional exhaustion due to the difficulty of allocating time between work, family, and personal activities. Under COR theory, this emotional strain represents acute resource depletion when individuals feel their pool of time and psychological energy is insufficient to meet competing demands (Hobfoll et al., 2018).

The emotional toll was further exacerbated by the “role spillover” effect, where conflicts in one role negatively impact another, such as fitness encroaching on family time. Huang et al. (2023) noted that this spillover leads to heightened feelings of guilt and reduced life satisfaction when social or family responsibilities are compromised. Additionally, participants reported the emotional impact of unmet expectations from family, work, or themselves. Unmet expectations, as discussed by P3, in these domains affected their sense of self-worth, a theme supported by research on the emotional consequences of persistent role conflict (Abdou et al., 2024). For mothers, “mommy guilt” was particularly relevant, with P8 reflecting on the challenges of balancing childcare with fitness, especially when her husband was unavailable. This emotional burden must be critically analysed through the lens of gendered social expectations and the “intensive mothering” ideology (Egami, 2025). In many South African households, patriarchal domestic norms persist, placing the primary responsibility of child-rearing and domestic mental load on women (Nel et al., 2024). While male participants framed exercise as an unproblematic act of self-improvement that enhanced their performance as a provider, female participants evaluated workout time against caregiving duties, internalizing guilt when personal leisure competed with domestic roles. The pressure of social comparison, particularly via social media, further amplifies these feelings. Moreover, P8 felt inadequate when comparing herself to other mothers, mostly on social media platforms, who quickly resumed fitness routines postpartum, reflecting research by Kirkpatrick and Lee (2022) that suggests social media fosters unrealistic expectations, contributing to negative emotional outcomes.

Participants in the study also experienced significant role conflict as they struggled to balance family, work and fitness commitments. Several described difficulties in prioritizing multiple responsibilities, with P3 noting that family and work often took precedence over fitness. This challenge is reflected in research by Aarntzen et al. (2019), who discuss “role overload,” where individuals face the need to sacrifice one role in favor of another. The inability to balance these responsibilities often results in frustration and feelings of inadequacy, further intensifying the experience of role conflict. The “time bind” theory (Damaske et al., 2014) also emerged as a relevant concept. It explores how individuals feel trapped by increasing work demands, which limits time for personal activities like fitness and negatively impacts well-being. As noted by P8, work commitments often overshadowed personal time, including gym sessions. This finding aligns with research by Huang et al. (2023), who found that work-related role demands frequently take priority over personal pursuits. The sacrifices participants made to maintain fitness routines also highlighted role conflict. The participant further described how childcare responsibilities sometimes interfered with fitness goals. Research by Martín-Rodríguez et al. (2024) found that consistently deprioritizing fitness due to work or family responsibilities can lead to negative psychological outcomes, including dissatisfaction and burnout. These experiences underscore the challenges of maintaining balance across competing life domains. The participants’ need to sacrifice certain roles in favor of others mirrors broader societal difficulties in managing multiple responsibilities, particularly in high-stress occupations or family roles.

Participants emphasized the critical role that family support plays in balancing fitness and personal commitments. For instance, P3 highlighted how shared priorities within families allow for mutual assistance, helping individuals manage both fitness and other responsibilities. This family collaboration is especially important during periods of intense training or personal obligations, as families often adjust schedules to accommodate each other’s needs. Research by An et al. (2024) supports these findings, showing that family support is linked to better health outcomes and reduced stress. Participants shared examples where partners took over household tasks to allow for fitness commitments, fostering a supportive environment that enhances overall family dynamics. Involving family in fitness activities was also seen to strengthen relationships and promote a healthier lifestyle. This approach aligns with Ha et al. (2019), who found that families engaging in physical activities together benefit from improved physical and emotional well-being.

However, family support alone may not always fully accommodate the demands of a structured training routine, particularly when family members do not share the same fitness goals or face competing home priorities (Jennings et al., 2018). While participants in this study possessed the flexibility to negotiate household roles, relying solely on domestic networks can create friction or fall short of meeting specific motivational needs. Consequently, when immediate family support reaches its practical limits, alternative external networks become essential for sustaining long-term training engagement.

This is precisely where the unique nature of CrossFit distinguishes itself from conventional physical activity. The CrossFit “box” is not merely a venue for exercise, but a specialized social system that generates high levels of subcultural social capital (Lautner et al., 2020). The CrossFit community emerged as another essential support system, providing motivation and accountability. For example, P4 mentioned that being part of this community made fitness more enjoyable and fostered a sense of belonging. Prochnow et al. (2022) noted that fitness communities like CrossFit encourage consistency through social support and group norms. Additionally, Leung et al. (2019) found that being part of a fitness community reduces exercise-related anxiety and enhances participants’ communication skills and social cohesion, all of which contribute to an improved fitness experience. Unlike individualistic fitness pursuits, CrossFit operates through small-group workouts, shared physical exertion, and peer encouragement. This communal environment normalizes high fitness commitment, providing participants with external validation and an accountability network that reinforces their identity as active individuals without the feeling of isolation (Lautner et al., 2020).

Friendships also played a role in sustaining fitness routines. For instance, P7 expressed how training with friends from CrossFit made the experience more enjoyable and helped maintain accountability. Social networks formed through these friendships provided essential support during challenging times, such as setbacks or injuries (Patterson et al., 2021). This camaraderie fosters a positive feedback loop that encourages continued participation in fitness activities (Scarapicchia et al., 2016).

The role of social support was highlighted as crucial for maintaining long-term fitness goals. As an illustration, P8 discussed the motivating influence of involving children in competitions, reflecting on how shared experiences can reinforce commitment. This is consistent with Ha et al.’s (2019) findings that encouragement from social networks is a significant factor in sustained fitness engagement. Encouragement from family and the CrossFit community helps individuals stay motivated, with verbal affirmations, participation in events, and presence at competitions fostering accountability. Social support also acts as a buffer against challenges in balancing fitness with other responsibilities. Participants described how having a supportive network helped them regain motivation when faced with personal, work, or fitness-related setbacks. Shahwan et al. (2024) emphasize that social support can enhance resilience, enabling individuals to overcome stress and burnout, which is crucial for sustaining long-term fitness goals. In COR theory terms, the CrossFit community operates as an external resource caravan (Hobfoll et al., 2018), replenishing social and emotional capital when work or family stress depletes personal resources. The findings from this research underscore the vital role that support systems play in balancing family, work, and fitness. The combined influence of family support, community engagement, and friendships creates a robust foundation for individuals to navigate their various life roles effectively. By leveraging these support systems, individuals can mitigate the emotional toll of role conflict and stress, fostering a healthier and more sustainable lifestyle. However, accessibility remains a concern, as CrossFit is often costly, restricting participation to those with the necessary financial resources. These findings align with existing literature and provide valuable insights into the experiences of CrossFit participants in managing these competing demands within the South African context.

In conclusion, this study explored how CrossFit participants in South Africa balance family, work, and fitness, identifying five key themes interpreted through COR theory. First, intentional planning and routine were critical for maintaining balance, with participants using early morning workouts, time blocking, and scheduling to prioritize fitness while reducing stress and fostering discipline. Second, physical exercise was essential for mental and physical well-being, with participants noting its role in stress management, energy boost, and personal growth, facilitated by the high physiological engagement and psychological detachment unique to CrossFit, though flexibility was necessary to avoid over-reliance. Third, flexibility in fitness routines was crucial for adapting to changing demands, with participants adjusting schedules and utilizing flexible class structures to maintain consistency. Fourth, an imbalance between family, work, and fitness domains often leads to guilt, role conflict, and emotional distress, which is deeply gendered and exacerbated by societal expectations of motherhood and role spillover. Finally, support systems, such as family, the CrossFit community, and friendships, played a pivotal role in sustaining long-term fitness goals by functioning as a source of subcultural social capital and resource replenishment. These findings highlight a number of challenges that participants experience regarding the balance of family, work, and fitness domains and also emphasize the importance of maintaining routine and flexibility and the utilization of social support in balancing multiple life domains to potentially foster well-being. The findings are, therefore, indicative of the need for future intervention-focused research in this regard.

## 6 Limitations and recommendations

The sample was predominantly white-African and the findings may not fully reflect the experiences of balancing these areas of life from other cultural backgrounds. Most participants did not have children. The findings may therefore not adequately capture the complexity of balancing parenting, work, and fitness responsibilities.

Given the limitations related to the sample regarding cultural diversity and the small number of participants with children, future research would benefit from expanding the geographical, socioeconomic, and cultural scope and including more participants with children.

In South Africa, where fitness activities such as CrossFit are often considered a luxury, it is important for future studies to explore how individuals from different socioeconomic backgrounds navigate the balance between family, work, and fitness. This could help highlight the barriers individuals face and offer insights into more inclusive approaches to fitness that can address these challenges.

Future research could explore various fitness modalities beyond CrossFit, which would offer alternative perspectives on how individuals navigate the balance between family, work, and fitness, which could yield valuable insights into the unique challenges and benefits associated with different fitness practices, ultimately enriching an understanding of the complex interplay between fitness and other life domains. Fitness modalities that are more affordable or adaptable to varying environments, such as home-based fitness or low-cost community fitness programs, may present viable alternatives in under-resourced communities.

Future studies should examine the specific mechanisms through which fitness communities, such as CrossFit, promote long-term engagement and motivation. Possibilities in this regard, for example, include assessing the relative impact of various types of support (emotional, practical, social) on maintaining fitness routines when faced with competing demands; exploring demographic comparisons to analyse how age, gender, and socioeconomic status influence individuals’ abilities to balance family, work, and fitness commitments.

Beyond guiding future academic inquiry, these empirical findings offer actionable, real-world strategies for employers, fitness facility owners, and wellness practitioners seeking to support individuals in navigating the work-family-fitness matrix. For employers and organizations, creating workplace environments that respect non-work temporal boundaries is critical. This can be operationalized by establishing policies that avoid mandatory early morning meetings or late-afternoon communications, thereby protecting the early morning time-blocks that employees rely on for stress recovery and exercise without experiencing work spillover. Furthermore, granting employees schedule autonomy through flexible working hours or hybrid arrangements enables them to adapt their personal health routines around acute work demands without triggering role strain. Corporate wellness packages can further support this by moving beyond standard gym discounts to subsidize structured, community-based or group fitness memberships that foster ongoing social capital and peer accountability.

For fitness facility owners and CrossFit affiliate managers, structural changes within the gym environment can significantly ease domestic and professional role conflicts. Facilities can optimize schedule flexibility by offering high-frequency early morning classes alongside penalty-free booking transfers to accommodate unpredictable work and family schedules. To alleviate caregiver guilt and domestic role friction—particularly for parents—facilities can introduce family-inclusive programming, such as concurrent youth classes, child-friendly waiting spaces during off-peak hours, or weekend family workouts. Additionally, intentional program design should leverage community dynamics through structured partner workouts and social events, reinforcing the fitness space as a supportive environment where personal health goals and relational identities align.

Finally, workplace wellness specialists, occupational health professionals, and industrial psychologists should reframe physical exercise not merely as a physical health obligation, but as an essential boundary management strategy for psychological detachment, emotional regulation, and resource recovery. Employee assistance programs and executive coaching can incorporate practical interventions focused on proactive time-budgeting, weekly calendar blocking, and establishing transparent boundary expectations across work, domestic, and personal life spheres to minimize guilt and emotional exhaustion.

## Data Availability

The raw data supporting the conclusions of this article will be made available by the authors, without undue reservation.
